# INPATIENT REHABILITATION AND FUNCTIONAL OUTCOME OF A CASE OF ANTI-SRP IMMUNE-MEDIATED NECROTIZING MYOPATHY

**DOI:** 10.2340/jrm-cc.v7.40653

**Published:** 2024-10-17

**Authors:** Nicole CHEN, Khin YAMIN THEIN, San SAN TAY

**Affiliations:** From the Department of Rehabilitation Medicine, Changi General Hospital, Singapore

**Keywords:** autoimmune disease, neurologic, myopathy, neurorehabilitation, rehabilitation outcome, robotics

## Abstract

**Objective:**

Immune-mediated necrotizing myopathy associated with anti-signal recognition particle antibodies is a rare and debilitating condition characterized by significant muscle weakness and resultant disability. Although there are existing recommendations for physical therapy and exercise for patients with myositis in current literature, effective rehabilitation guidelines for such patients have not been fully established.

**Case report:**

A 42-year-old woman presented with subacute onset proximal upper and lower limb weakness and dysphagia. She was diagnosed with anti-SRP immune-mediated necrotizing myopathy and was promptly initiated on aggressive immunosuppressive therapy. Despite this, she had significant impairment in function, including the inability to ambulate.

**Intervention and outcome:**

The patient underwent an intensive structured inpatient rehabilitation programme consisting of strength and endurance exercises combined with functional exercises and robot-assisted gait training. This resulted in major improvements in objective outcomes including Functional Independence Measure, Modified Barthel Index, 10-metre-walk test and 6-minute-walk test scores. The programme was well tolerated by the patient with no adverse events.

**Conclusion:**

This case details the crucial role of a structured rehabilitation programme in the holistic management of a patient with anti-SRP immune-mediated necrotizing myopathy. It also highlights the use of robotics in gait training, resulting in successful functional outcomes for the patient.

## Lay Abstract

Immune-mediated necrotizing myopathy (IMNM) associated with anti-signal recognition particle (anti-SRP) antibodies is a rare form of myositis characterized by significant muscle weakness and resultant disability. Effective management requires a comprehensive approach, including pharmacological treatment and multidisciplinary rehabilitation. Here, we present a case of a 42-year-old woman who presented with a 3-month history of proximal upper and lower limb weakness and difficulty swallowing. She was diagnosed with anti-SRP IMNM and was promptly initiated on aggressive immunosuppressive therapy. Despite this, she had significant impairment in function, including the inability to ambulate. The patient underwent a structured inpatient rehabilitation programme including robot-assisted gait therapy, resulting in significant improvement in her symptoms and a desirable functional outcome. This case highlights the crucial role of a structured rehabilitation programme in the holistic management of such patients.

Idiopathic inflammatory myopathies (IIM) are a heterogenous group of autoimmune disorders which can be classified into 4 major subgroups: dermatomyositis (DM), immune-mediated necrotizing myopathy (IMNM), anti-synthetase syndrome and inclusion body myositis (IBM) (1). At present, the 2 main subtypes of IMNM include anti-signal recognition particle (anti-SRP) associated IMNM, and anti-3-hydroxy-3-methylglutaryl-coA reductase (anti-HMGCR) associated IMNM (2). The IIMs are rare entities, with incidence rates estimated to be between 0.2 and 2 per 100,000 person-years and prevalence between 2 and 25 per 100,000 people (3).

Anti-SRP associated IMNM is characterized clinically by subacute to chronic symmetrical proximal muscle weakness and elevated creatine kinase (CK) levels (4). Therapeutic management typically involves a combination of immunosuppressive therapy and supportive care. The 224th ENMC International Workshop (5) reported that anti-SRP IMNM is one of the most disabling auto-immune myopathies, with poor muscle recovery and resultant sustained disability even with treatment. Although there are existing recommendations for physical therapy and exercise for patients with myositis in the current literature, effective rehabilitation guidelines for such patients have not been fully established. Rehabilitation interventions focusing on muscle strengthening, endurance training, and functional activities are essential for optimizing outcomes in these individuals. This case report details the inpatient rehabilitation course and highlights the potential of innovative rehabilitation technologies in optimizing outcomes for individuals with IMNM and other neuromuscular disorders.

## CASE REPORT

A 42-year-old woman with no relevant past medical history presented to our Emergency Department with a 3-month history of progressive proximal upper and lower limb weakness associated with dysphagia. Physical examination revealed Medical Research Council (MRC) grade 2 power of bilateral hip flexion and grade 3 power of bilateral hip extension. Shoulder abduction (MRC 3) and neck flexion (MRC 2) were also weak. Power was otherwise full and sensation was intact. Negative Inspiratory Force was -60 cm H_2_0, showing no evidence of respiratory muscle weakness. Laboratory investigations showed markedly elevated CK levels (8890 U/L) and normal inflammatory markers. Anti-SRP antibodies were present. Electromyography revealed myopathic changes, and muscle biopsy demonstrated necrotic fibres with minimal inflammation, consistent with IMNM. She was referred to the Neurology team who made the diagnosis of anti-SRP IMNM.

In terms of extra-muscular manifestations, there was no evidence of cardiomyopathy based on transthoracic echocardiogram that was done. A thorough malignancy screen was negative. There were 2 episodes of per-rectal bleeding and a colonoscopy was done. This revealed several large rectal ulcers which were biopsied. Histology was in keeping with features of acute inflammatory cell infiltrate with no granuloma, dysplasia or malignancy. The patient opted for conservative management of these ulcers. No further bleeding occurred.

**Fig. 1:**
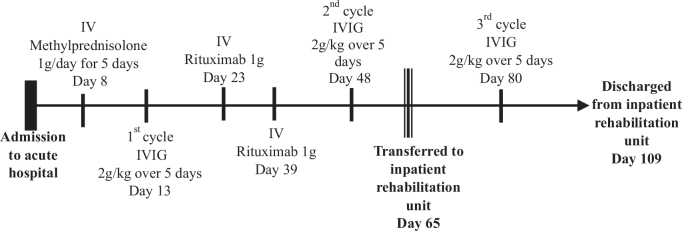
Summary of Immunosuppressive therapy. (IV: intravenous; IVIG: intravenous immunoglobulin).

On day 8 of admission, the patient was initiated on intravenous methylprednisolone 1 g daily for 5 days. This was immediately followed by a course of intravenous immunoglobulin (IVIG) 2 g/kg over 5 days in view of persistent weakness. Despite this, there was no improvement in power. The patient was thus given Rituximab 1 g on day 23 and again on day 39 of admission, as per our hospital protocol. Figure 1 provides a detailed summary of the immunotherapy regime received by the patient. She was placed on a slow tapering regimen of oral prednisolone, starting at 60 mg/day, then reducing by 10 mg every 30 days. At discharge from the acute hospital (day 109), she was on prednisolone 20 mg/day.

Following stabilization, the patient was transferred to our inpatient rehabilitation unit on day 65 of admission. Upon transfer, she still had significant neck and proximal limb weakness as detailed in Table I. She had poor truncal control and required maximal assistance with bilateral upper limb support to stand. She required moderate assistance in all her activities of daily living (ADL). The patient was assessed by the speech therapist to have mild oropharyngeal dysphagia characterized by slow mastication, delayed effortful swallows and reduced laryngeal excursion. She reported fatigue effect especially over feeding on solids and was placed on a minced and moist diet.

**Table I:** Manual muscle testing on admission and at discharge from inpatient rehabilitation unit (as per MRC grade)

Movement tested	At presentation to the emergency department Day 1	Admission to rehabilitation unit Day 65	Discharge from rehabilitation unit Day 109
Neck Flexion	2	2	4
Shoulder abduction	3	3	5
Grip	3	4	5
Hip flexion Hip extension	2 2	2 3	5 5
Ankle dorsiflexion	3	3	5

IVIG: Intravenous immunoglobulin; MRC: Medical Research Council.

On attempting to ambulate, she required the use of a platform rollator and maximal assistance by the therapist. She fatigued quickly, managing only to mobilise 5 m. She had a stooped posture due to truncal extension weakness, reduced hip flexion during swing phase and reduced hip and knee extension during midstance. The patient was also extremely fearful of falling.

The rehabilitation programme consisted of daily physical therapy focusing on muscle strengthening and endurance exercises. These initially consisted of isometric body weight exercises in particular to strengthen her proximal and core muscles, then progressed to resistance exercises with elastic bands and light weights. Table II shows the inpatient rehabilitation physical therapy protocol in detail.

**Table II:** Detailed inpatient rehabilitation physical therapy protocol

Phase of Rehabilitation	Phase 1: Prior to admission to rehabilitation ward Day 1 to Day 64	Phase 2: Inpatient rehabilitation ward Day 65 to Day 80	Phase 3: Inpatient rehabilitation ward (post 3^rd^ cycle IVIG) Day 81 to Day 109
Muscle strengthening/endurance exercises	Bed exercises: heel slides 10 repetitions × 2 sets ankle pumps 10 repetitions × 2 sets isometric quadricep exercises 10 repetitions, 3 second hold	Bed exercises: straight leg raises 10 repetitions × 2 sets double leg bridging 8 repetitions × 2 sets cervical extension in supported sitting 3 repetitions × 2 sets clamshells 10 repetitions × 2 sets each side Sit to stand exercises with platform rollator 10 repetitions × 3 sets Standing alternate hip flexion 10 repetitions × 2 sets Marching on the spot with platform rollator 10 repetitions × 3 sets Recumbent cross trainer 15 min, 0 resistance, light-moderate intensity	Chest presses with pillow 10 repetitions Biceps curls with pillow 10 repetitions Overhead tricep extensions 10 repetitions Sit to stand exercises with walking frame 10 repetitions × 3 sets Alternate stepping on 6 inch step 10 repetitions × 2 sets
Resistance exercises	Nil	Resisted shoulder presses 10 repetitions Manual resisted leg presses in sitting 10 repetitions	Pneumatic leg press machine; glutes bias with resistance band around thigh 20kg bilaterally 8 repetitions × 3 sets Recumbent cross trainer 10 min, level 3 resistance, moderate intensity
Balance/core exercises	Static sitting for 1 min, progressing to 3 min supported	Static sitting × 30 seconds unsupported, progressing to 3 min Reach anteriorly for railing and return to upright sitting × 10 repetitions × 2 sets Reach laterally to right, then left 10 repetitions each × 1 set	Supine knee rolls activating obliques 10 repetitions × 3 sets Reclined sit ups 10 repetitions × 2 sets Crunches 10 repetitions × 3 sets

In view of the patient’s poor trunk control, proximal weakness and increased fears of falling limiting conventional rehabilitation approaches, our team decided to embark on the use of robot- assisted gait training (RAGT) during the second week of her inpatient rehabilitation course. Our team utilized a patient-guided suspension system (Andago^TM^) consisting of overhead support bars attached to a harness worn by the patient. It provides customisable weight support and minimises the risk of falls with its safety harness. During her first session, the patient was able to ambulate 195 m in a 30-min session with 2 rest stops in between. She was provided with 10 kg weight support during that session. The weight support and the harness straps assisted her in assuming a more upright truncal position during RAGT. This greatly motivated her and gave her confidence to aim for higher ambulation goals. After 3 sessions that occurred over a week, her trunk control improved considerably and she gained enough confidence to attempt overground ambulation with a rollator frame. She subsequently underwent a combination of overground ambulation training and adjunct RAGT. She completed a total of 19 sessions of RAGT over 4 weeks and was able to ambulate 735 m in a 30-min session during her final RAGT training. Prior to discharge, she was able to ambulate with a walking stick and close supervision for 300 m.

Occupational therapy integrated the use of Proprioceptive Neuromuscular Facilitation (PNF) techniques in ADL retraining and educated the patient on energy conservation techniques, pacing strategies and fall recovery strategies. She was modified independent in her basic ADL but still required supervision in her instrumental ADL prior to discharge.

Speech therapy consisted of tongue range of motion and strengthening exercises along with mylohyoid isometric exercises. The patient had meals in an upright seated position with head support in view of fatigue over the meal. She was taught paced feeding with intermittent rest breaks as required. Her swallowing improved and she was able to tolerate a normal diet prior to discharge. Functional Oral Intake Scale (FOIS) improved from 5 to 7.

Over the course of the 6-week rehabilitation programme, the patient showed significant improvements in muscle strength, mobility and functional independence. Other outcome measures including manual muscle testing, functional independence measure (FIM), 10 metre-walk test (10MWT) and 6 min-walk test scores demonstrated marked improvements (Table III). The patient reported substantial reduction in fatigue as evidenced by scores on the fatigue severity scale (FSS), and also greater self-confidence in her abilities to self-care. She was provided with a customized self-exercise programme to maintain gains achieved during rehabilitation and facilitate long-term adherence to exercise. She spent a further 2 weeks at a community hospital to work on community ambulation, in particular to navigate stairs and ramps, prior to her discharge home.

**Table III:** Relevant functional assessments on admission and at discharge from inpatient rehabilitation unit

Outcome measures	Admission to rehabilitation unit Day 65	Discharge from rehabilitation unit Day 109
Transfer	Maximum assistance	Independent
Sit-to-stand	Maximum assistance/Dependent	Independent
Ambulation	Unable	Supervision with walking stick
10MWT	Unable	0.77 m/s
6mWT	Unable	360 m
FIM total	63	110
FIM motor FOIS FSS	32 5 54	79 7 19
MBI	23	95

10MWT: 10-metre walk test; 6mWT: 6 minute walk test; FIM: Functional Independence Measure; FOIS: Functional Oral Intake Scale; FSS: Fatigue Severity Scale; MBI: Modified Barthel Index.

A month later at the outpatient clinic, there were sustained improvements in muscle strength. The patient reported being able to independently perform her basic and instrumental ADL and was considering the possibility of returning to employment.

## DISCUSSION

Anti-SRP IMNM poses significant therapeutic challenges due to its heterogenous clinical presentation and variable treatment response. Immunomodulation remains the cornerstone of medical treatment in IMNM. However, despite aggressive immunotherapy, many patients have persistent weakness and disability. Watanabe et al. (6) reported that a third of patients with anti-SRP IMNM from the Japanese registry had persistent residual disability as evidenced by Modified Rankin Scale scores of 3–5. This was similarly reported by Tan et al. (7), who performed a cohort study on a multi-ethnic Malaysian cohort. Although medical treatment has been relatively well described, literature on the rehabilitative care of patients with IMNM is scarce. We attempt to describe the holistic rehabilitation approach for a patient with IMNM.

Physical therapy plays a crucial role in optimizing functional outcomes in patients with anti-SRP IMNM. Historically, patients with IIM were refrained from strenuous physical activity for fear of worsening muscle damage. In recent times, numerous studies have described the positive effects of aerobic and/or resistance exercise in such patients in helping to maintain or improve muscle strength, aerobic fitness and functional measures. In their narrative review, Varone et al. (8) reported that a combination of aerobic and resistance training performed twice a week increased autophagy, aiding in skeletal muscle repair, while also reducing the expression of genes associated with muscle atrophy. The authors suggested that patients should be encouraged to participate in aerobic activities more frequently than twice a week. Additionally, resistance training twice a week, with 8–12 repetitions at maximum effort, has been shown to reduce muscle atrophy and intramuscular lipid deposits, as confirmed by follow-up muscle biopsies. A systematic review by Thillo et al. (9) concluded that physical therapy is safe in individuals with idiopathic IIM in both active and quiescent disease states. It also reported that addition of resistance training is safe, although no clear conclusion on effect could be drawn.

Further, a narrative review by Ramdharry et al. (10) examining exercise in individuals with idiopathic IIM found that exercise is safe, based on various measures of disease activity, serum CK levels, and indicators of inflammation, pain, or fatigue. Some studies using aerobic exercise protocols reported improvements in cardiorespiratory fitness and exercise capacity. Resistance training also showed enhancements in muscle function across all studies included in the review, assessed through manual muscle testing, 1-repetition maximum tests, and quantitative evaluations using handheld dynamometry as well as isokinetic and isometric dynamometry.

To ensure safe and effective rehabilitation while minimising the risk of exacerbating muscle weakness and inflammation, our team initiated the patient on low-intensity exercises and gradually increased exercise intensity and duration in small increments. This was done based on the patient’s tolerance and according to her comfort level. Strengthening exercises focused on low-resistance, high-repetition sets of exercises targeting weak muscle groups. The use of bodyweight exercises, resistance bands and light weights helped avoid excessive strain on muscles. Our team also incorporated functional exercises that mimic ADL to improve muscle coordination, balance and mobility. The patient was allowed adequate rest periods between therapy sessions to prevent muscle fatigue and promote recovery. She was monitored closely during and after therapy sessions for signs of muscle weakness, fatigue, pain, or exacerbation of symptoms, and the rehabilitation programme was adjusted as necessary.

Traditional rehabilitation interventions for gait training were hindered by patient’s fatigue, poor effort tolerance and fear of falling. The use of adjunctive robot-assisted gait training offers several advantages in the rehabilitation of patients with IMNM. Firstly, the patient was initially offloaded 15% of her total body weight by having weight support of 10 kg. This reduction in load enabled greater ambulation distance, allowing increased repetition and building endurance. In her first session on the patient-guided suspension system, she was able to walk 39 times more than what she managed without. Next, the harness provided support and stability, which assisted in boosting the confidence of the patient and help her overcome the fear of falling. This played an important role in motivating her, leading to greater participation and adherence to therapy sessions. A study on the utilization of Hybrid Assistive Limb (HAL) device on slowly progressive rare neuromuscular diseases showed that it was safe and effective (11). Some of these neuromuscular diseases present with proximal weakness and fatigability similarly found in anti-SRP IMNM. In addition to using the HAL, the patients were also supported using a mobile harness in their training. The availability of the type of robotic device would vary from centre to centre. A device with a harness system would be helpful with patients with anti-SRP IMNM.

Fatigue is a prevalent and debilitating symptom in IMNM, often affecting patients’ ability to participate fully and progress in rehabilitation. A systematic review by Misse et al. (12) reported that studies available in recent literature revealed controversial results regarding the effects of exercise training on chronic fatigue in patients with systemic autoimmune myopathies. In this case, a multidisciplinary approach was used to manage the patient’s fatigue which featured prominently during her inpatient rehabilitation course. Apart from the use of robot-assisted gait training, therapy sessions were structured to accommodate fluctuations in energy levels. Exercise duration and load were gradually increased with time, as tolerated by the patient. Psychological support in the form of counselling to address the patient’s anxiety was also provided.

In conclusion, we report a case detailing the inpatient rehabilitation course and functional outcome of a patient with anti-SRP IMNM. To our knowledge, the use of a patient-guided suspension system in the rehabilitation of IMNM has not been reported in the literature. In this case, the adjunctive use of the patient-guided suspension system resulted in good functional outcome and walking ability of the patient. Other forms of robot-assisted gait training could potentially similarly benefit patients with IMNM and other neuromuscular conditions. Further research is needed to validate the efficacy and safety of robot-assisted gait training in a larger cohort of IMNM patients/patients with neuromuscular conditions. This case underscores the importance of a multidisciplinary approach in the comprehensive management of anti-SRP IMNM. Further research is warranted to elucidate optimal treatment strategies, establish standardized rehabilitation protocols for IMNM patients, and evaluate their long-term outcomes.
